# Residential Radon Exposure and Incidence of Childhood Lymphoma in Texas, 1995–2011

**DOI:** 10.3390/ijerph121012110

**Published:** 2015-09-25

**Authors:** Erin C. Peckham, Michael E. Scheurer, Heather E. Danysh, Joseph Lubega, Peter H. Langlois, Philip J. Lupo

**Affiliations:** 1 Department of Pediatrics, Section of Hematology-Oncology, Baylor College of Medicine, One Baylor Plaza, MS BCM305, Houston, TX 77030, USA; E-Mails: Erin.Peckham@bcm.edu (E.C.P.); Scheurer@bcm.edu (M.E.S.); Danysh@bcm.edu (H.E.D.); Lubega@bcm.edu (J.L.); 2 Birth Defects Epidemiology and Surveillance Branch, Texas Department of State Health Services, MC 1964, P.O. Box 149347, Austin, TX 78714-9347, USA; E-Mail: Peter.Langlois@dshs.state.tx.us

**Keywords:** childhood cancer, epidemiology, lymphoma, residential radon, Texas Cancer Registry

## Abstract

There is warranted interest in assessing the association between residential radon exposure and the risk of childhood cancer. We sought to evaluate the association between residential radon exposure and the incidence of childhood lymphoma in Texas. The Texas Cancer Registry (*n* = 2147) provided case information for the period 1995–2011. Denominator data were obtained from the United States Census. Regional arithmetic mean radon concentrations were obtained from the Texas Indoor Radon Survey and linked to residence at diagnosis. Exposure was assessed categorically: ≤25th percentile (reference), >25th to ≤50th percentile, >50th to ≤75th percentile, and >75th percentile. Negative binomial regression generated adjusted incidence rate ratios (aIRR) and 95% confidence intervals (CI). We evaluated lymphoma overall and by subtype: Hodgkin (HL; *n* = 1248), Non-Hodgkin excluding Burkitt (non-BL NHL; *n* = 658), Burkitt (BL; *n* = 241), and Diffuse Large B-cell (DLBCL; *n* = 315). There was no evidence that residential radon exposure was positively associated with lymphoma overall, HL, or BL. Areas with radon concentrations >75th percentile had a marginal increase in DLBCL incidence (aIRR = 1.73, 95% CI: 1.03–2.91). In one of the largest studies of residential radon exposure and the incidence of childhood lymphoma, we found little evidence to suggest a positive or negative association; an observation consistent with previous studies.

## 1. Introduction

In the United States (US), lymphoma is the third most frequently occurring form of cancer among children, representing approximately one-third of all malignancies in those less than 20 years of age [1]. The overall incidence of lymphoma in children and adolescents is approximately 23 cases per million, which varies by age and subtype [1,2]. Due to advancements in therapy, the overall 5-year survival rate for those diagnosed with lymphoma is greater than 85% [3,4]. However, survivors experience severe and often life-long health consequences as a result of their treatment, including an elevated risk of second primary cancers and other serious chronic conditions [5,6,7,8,9]. Because of these often devastating late effects, the identification of risk factors for the prevention of childhood lymphoma is critical [10]. While environmental pollutants have long been suspected to play a role in the development of childhood lymphoma [11,12], very few risk factors have been identified and confirmed. In fact, most cases of this childhood malignancy are of unknown etiology [13,14,15].

Radon-222 and its decay products are classified as Group 1 carcinogens by the International Agency for Cancer Research [16]. This radioactive gas, resultant from the chemical breakdown of uranium, is associated with severe health outcomes including lung cancer, making exposure to this environmental hazard a significant public health risk [17,18,19]. Radon poses a particular problem as it is an odorless, colorless, naturally-occurring gas that tends to accumulate in homes, often remaining undetected by those exposed [20,21]. The ionizing radiation emitted from the radioactive decay of radon can lead to DNA damage and influence other biologic mechanisms, which may ultimately induce tumorigenesis [22]. Given the established association between radon exposure and lung cancer [23,24,25], there is well-founded interest in evaluating the association between residential exposure to radon and the risk of other malignancies, especially those occurring in children [26,27,28,29]. Previous studies have been largely equivocal in relation to childhood leukemia, and very few assessments have investigated lymphoma specifically [27,28,30,31], although exposure to high levels of ionizing radiation is suggested to increase the risk of non-Hodgkin lymphoma (NHL) among children [12]. As hematologic malignancies including lymphoma arise from mutated cells in the immune system and bone marrow, they are considered one of the most radiosensitive cancers [32,33]. As such, further elucidation of the role radon may play in childhood lymphoma is warranted.

Therefore, we sought to determine if there is an association between indoor radon exposure and the incidence of childhood lymphoma in Texas, a state characterized by variable concentrations of radon due to its diverse geology and history of uranium mining [34]. Additionally, Texas is home to one of the world’s largest population-based cancer registries and was part of the US Geological Indoor Radon Survey, which included mapping of key radon zones in the state [34,35].

## 2. Experimental Section

### 2.1. Study Population

Lymphoma cases were obtained from the Texas Cancer Registry (TCR) and limited to those diagnosed less than 20 years of age (*n* = 2147) for the period of 1995–2011. The TCR is one of the world’s largest, statewide and nationally certified population-based cancer registries, and is ranked “high-quality” by the Centers for Disease Control [36]. The selection of lymphoma subtypes in this study was based on the most recent version of the International Classification of Childhood Cancer (ICCC) [37] and ICD-O3 histologic codes (ICD-O3 HC), in conjunction with World Health Organization 2008 site groups (WHO 2008 SG). For the purposes of this study, we grouped the lymphoma subtypes as follows: Hodgkin (HL; ICD-O3 HC: 9650–9655, 9659, 9661–9665, 9667 and WHO 2008 SG: 33011, 33012; *n* = 1248); Non-Hodgkin, excluding Burkitt (non-BL NHL; ICD-O3 HC: 9590 (NHL Unspecified); 9591 (NHL B-cell not otherwise specified (NOS)); 9670–9673, 9823 (NHL Mature B-cell-Chronic Small); 9679–9680, 9684 (NHL Mature B-cell-Diffuse Large (DLBCL)); 9698, 9690, 9691 (NHL Mature B-cell-Follicular); 9689, 9699 (NHL Mature B-cell-Nodal Marginal Zone); 9675 (NHL Unknown lineage, NOS); 9727, 9728, 9811 (NHL Precursor-B-cell) and WHO 2008 SG: 33041, 33042; *n* = 658); Burkitt (BL; ICD-O3 HC: 9687 and WHO 2008 SG: 33041, 33042; *n* = 241); and DLBCL alone (ICD-O3 HC: 9679-9680, 9684 and WHO 2008 SG: 33041, 33042; *n* = 315). Population estimates were obtained from the 2000 US Census for all children less than 20 years of age in Texas at that time-point (*n* = 6,523,632). The Institutional Review Boards (IRB) at Baylor College of Medicine and the Texas Department of State Health Services approved this study.

### 2.2. Exposure Assessment

Exposure to radon was estimated using data from the Texas Indoor Radon Survey, which was a statewide assessment of indoor residential radon conducted January through March, 1991 and supported by a grant from the US Environmental Protection Agency (EPA) [34,35]. Details of the Texas Indoor Radon Survey have been described previously [34,35]. Briefly, residential, owner-occupied single family dwellings were randomly selected and contacted from telephone lists. As Texas is a large state with the potential for regional variation in radon levels given a diverse geologic history, the random allocation of radon detectors used a regional sampling plan, where all counties were grouped into regions based on plausible indoor radon exposure. As example, the Panhandle area of Texas has been shown to have the highest potential for high radon levels, and previously has been the only region in Texas to report a sizable number of homes with residential radon levels greater than 148 Bq/m^3^, the threshold of concern per EPA guidelines [34]. Additionally, other regions throughout the state, such as Llano Uplift, the Big Bend area, and South Texas also have potential for elevated radon levels given the underlying geologic substructure. In order to determine the study regions for the radon measurements, survey staff grouped all Texas counties together with respect to their potential for residential radon based on subsurface geologic and population data. Then, contiguous counties with similar residential radon potentials were grouped into regions. Further, taking into consideration the potential for rural areas to have less survey representation, large metropolitan areas (e.g., Harris county and the Dallas/Fort-Worth area) were designated as their own regions which were then sampled at a lower percentage than rural areas, thus ensuring that less-populated regions would have adequate survey sample size (Table 1). Thus, in constructing the regions for measurement the goal was two-fold: (a) have regions comprised of homogenous residential radon levels within each region; and (b) ensure a balance in the proportion of houses sampled from both metropolitan and rural areas. Thirteen relatively homogenous regions with little within-region radon variability were thus defined based on estimated radon levels, and all residents within a specific region had an equal chance of being contacted via telephone for survey participation. Notably, Texas had the largest number of regions in comparison to any other state surveyed under the EPA sponsored program, and offered a comprehensive overview of residential radon levels across Texas.

**Table 1:** Description of the thirteen study regions of the The Texas Indoor Radon Survey, 1991.

Region Name	Region Number	Measurements for Analysis, *n*
Southwest Texas	1	208
El Paso	2	97
Big Bend	3	122
West Texas Shales	4	241
North Texas	5	348
Dallas/Fort Worth	6	172
East Texas	7	296
Llano Uplift	8	213
Central Texas (Austin-San Antonio)	9	237
Tertiary Sands Crescent	10	204
Harris County (Houston)	11	122
Gulf Coast	12	215
Texas Panhandle	13	258

The randomly selected residents were then asked to place an activated charcoal adsorption canister in a centrally-located, interior room for the course of seven days. The charcoal absorbed radon decay products which subsequently produced gamma rays able to be measured by scintillation detectors. Upon completion of the seven days, participants sealed the canister and immediately returned via mail to the US Environmental Protection Agency laboratory, where the gamma ray quantification took place. Over seventy-percent of the 4031 canisters sent out (*n* = 2890; 71.7%) were returned and contributed to the present analysis [35]. The arithmetic mean radon concentration for each region (picoCuries/liter; pCi/l), as published in and available from the final survey report, was assigned to each study subject based on either (i) the county of residence at time of lymphoma diagnosis for cases or (ii) county of residence at time of the 2000 Census for population estimates. For this analysis, exposure was assessed with the unit of comparison as the mean radon concentration in each of the thirteen geologic regions, converted from pCi/l to Becquerel per cubic meter (Bq/m^3^) to be consistent with international nomenclature.

### 2.3. Covariate Selection

We considered age at diagnosis, sex, and race/ethnicity, as well as county-level socioeconomic status (SES) and urbanization as potential confounders. Age at diagnosis was assessed categorically with four levels (0–4, 5–9, 10–14, and 15–19 years of age). Race/ethnicity was categorized as non-Hispanic white, Hispanic white, non-Hispanic black, and other which included those who reported being Asian, American Indian/Alaska Native, Hawaiian Island/Pacific Islander, some other race alone, and two or more races. Hispanics of one or more races were classified as “two or more races”, and counted in the “other” category. County-level SES and urbanization were based on data from the 2000 Census, with both case and denominator data for SES and urbanization assigned based on county of residence at diagnosis or at the time of the 2000 Census. Low SES counties were defined as those with greater than the median proportion of the population living under the Federal Poverty Level. Urban counties were defined as those with greater than or equal to 50% of the population living in an “Urban” area as defined by the US Census. County-level SES and urbanization were assessed categorically in four levels as urban-high SES, rural-high SES, urban-low SES, and rural-low SES. No cases were missing data on our covariates of interest.

### 2.4. Statistical Analyses

Negative binomial regression was used to explore the association between residential radon exposure and the incidence of childhood lymphoma, with our unit of comparison being the mean radon concentration in each of the thirteen geologic regions. While Poisson regression is a traditional method to analyze count data such as this, at times the assumptions of this model are violated as individual counts are more variable, or overdispersed, than is accommodated under the Poisson distribution [38,39]. This limitation may be overcome through the use of a negative binomial model as a random term reflects unexplained between-subject differences to account for overdispersion of data [40]. Thus, given the overdispersed nature of our data, modeling under negative binomial regression was statistically more appropriate than the utilization of Poisson regression [41].

We conducted analyses among all lymphoma cases per geologic region, and separately for each of the following lymphoma subtypes: HL, non-BL NHL, BL, and DLBCL. Mean radon concentration in each region was the primary exposure, and other independent variables included in the model were county-level SES and level of urbanization, sex, age group, and race/ethnicity. Denominator data were obtained from the 2000 Census for all 6.5 million children less than 20 years of age in Texas. We generated unadjusted incidence rate ratios (IRR) and 95% confidence intervals (CI), and IRR adjusted for sex, race/ethnicity, age group, and county-level SES/urbanization (aIRR).

**Figure 1:**
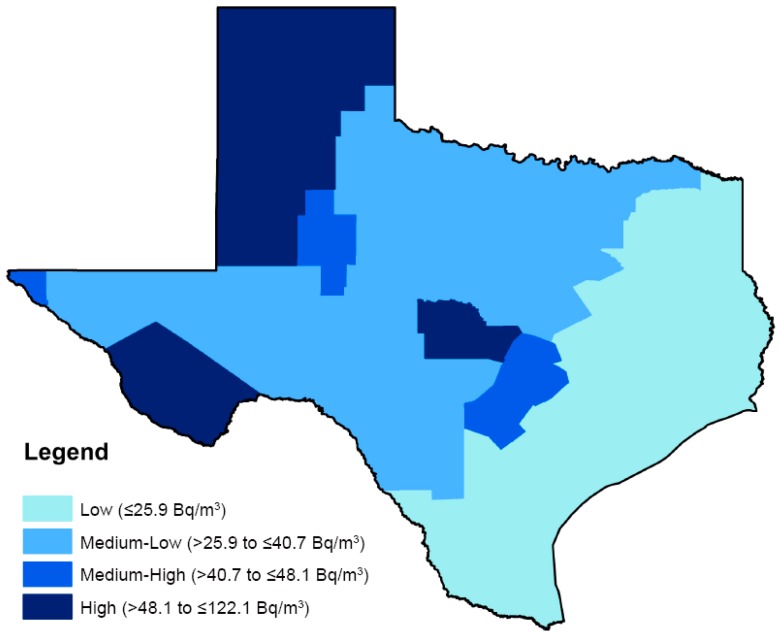
Quartile distribution of residential radon exposure from The Texas Indoor Radon Survey, 1991.

In order to evaluate incidence of childhood lymphoma, regional mean radon concentrations were assessed categorically comparing the regions with “medium-low” radon concentrations (>25th to ≤50th percentile; >25.9 to ≤40.7 Bq/m^3^), “medium-high” radon concentrations (>50th to ≤75th percentile; >40.7 to ≤48.1 Bq/m^3^), and the “highest” radon concentrations (>75th percentile; >48.1 Bq/m^3^) to regions with the “lowest” radon concentrations (≤25th percentile; ≤25.9 Bq/m^3^). Cut-points were based on the distribution across the state (Figure 1). All analyses were conducted using STATA (version 13, StataCorp, College Station, TX, USA).

## 3. Results and Discussion

### 3.1. Results

Demographic characteristics of the lymphoma cases diagnosed under the age of 20 in Texas between 1995 and 2011 are shown in Table 2. Lymphoma cases in our study population were predominantly diagnosed between the ages of 15–19 years (51.2%), male (61.2%), non-Hispanic white (47.9%), and lived in urban, high SES counties (66.2%). Texans under 20 years of age were distributed evenly with respect to age and sex, and as in lymphoma cases, most were non-Hispanic white (42.8%) and the majority lived in urban, high SES counties (66.3%).

The distribution of mean residential radon concentrations measured in Bq/m^3^ across Texas is presented in Table 3. The arithmetic mean radon concentration across regions was 45.97 Bq/m^3^, with levels ranging from 9.25 Bq/m^3^ to 122.10 Bq/m^3^. The median radon concentration was 40.70 Bq/m^3^.

**Table 2:** Demographic characteristics of lymphoma malignancy subtypes among children and adolescents <20 years of age diagnosed in Texas, 1995–2011.

Characteristic	All Lymphomas (*n* = 2147)	Hodgkin Lymphoma (*n* = 1248)	NHL Excluding BL (*n* = 658)	Burkitt Lymphoma (*n* = 241)	Diffuse Large B-Cell Lymphoma (*n* = 315)	Total TX Population <20 Years Old * (*n* = 6,523,632)
Age of Diagnosis (years), *n* (%)						
<5	197 (9.2)	38 (3.0)	110 (16.7)	49 (20.3)	49 (20.3)	1,610,302 (24.7)
5 to <10	316 (14.7)	117 (9.4)	123 (18.7)	76 (31.5)	76 (31.5)	1,660,902 (25.5)
10 to <15	534 (24.9)	323 (25.9)	148 (22.5)	63 (26.1)	63 (26.1)	1,642,973 (25.2)
15 to <20	1100 (51.2)	770 (61.7)	277 (42.1)	53 (22.0)	53 (22.0)	1,609,455 (24.7)
Sex, *n* (%)						
Male	1314 (61.2)	702 (56.3)	404 (61.4)	208 (86.3)	208 (86.3)	3,348,530 (51.3)
Female	833 (38.8)	546 (43.8)	254 (38.6)	33 (13.7)	33 (13.7)	3,175,102 (48.7)
Race/Ethnicity, *n* (%) **						
Non-Hispanic White	1029 (47.9)	590 (47.3)	310 (47.1)	129 (53.5)	129 (53.5)	2,790,778 (42.8)
Hispanic White	787 (36.7)	447 (35.8)	261 (39.7)	79 (32.8)	79 (32.8)	1,484,094 (22.7)
Non-Hispanic Black	243 (11.3)	158 (12.7)	61 (9.3)	24 (10.0)	24 (10.0)	820,640 (12.6)
Other ^†^	88 (4.1)	53 (4.3)	26 (4.0)	9 (3.7)	9 (3.7)	1,428,120 (21.9)
County-level SES/Level of Urbanization ^†^, *n* (%)						
Urban, Higher SES	1421 (66.2)	818 (65.5)	434 (66.0)	169 (70.1)	169 (70.1)	4,326,761 (66.3)
Urban, Lower SES	510 (23.8)	296 (23.7)	167 (25.4)	47 (19.5)	47 (19.5)	1,520,114 (23.3)
Rural, Higher SES	144 (6.7)	86 (6.9)	37 (5.6)	21 (8.7)	21 (8.7)	425,312 (6.5)
Rural, Lower SES	72 (3.4)	48 (3.9)	20 (3.0)	4 (1.7)	4 (1.7)	251,445 (3.9)
Mean Residential Radon Concentration (Bq/m^3^)	33.3	32.4	34.3	34.8	35.3	-----

SES, Socioeconomic status; TX, Texas; * Source: 2000 U.S. Census, U.S. Census Bureau; ** “Other” Race/Ethnicity includes, those who reported being Asian, American Indian/Alaska Native, Hawaiian Island/Pacific Islander, Some other race alone, and Two or more races. ^†^ Lower SES defined as counties with greater than the median proportion of the population living under the Federal Poverty Level; Urban defined as those counties with greater than or equal to 50% of the population living in Census defined “Urban” county.

**Table 3:** Distribution of residential radon exposure from The Texas Indoor Radon Survey, 1991.

Indoor Radon Concentration (Becquerel per Cubic Meter; Bq/m^3^)	
Arithmetic mean	45.97 *
Geometric mean (95% CI)	37.75 (25.19–56.58)
Minimum	9.25
Maximum	122.10
Percentile	
25th	25.90
50th	40.70
75th	48.10
90th	96.20
99th	122.10

* Mean radon concentration levels as measured in Bq/m^3^ for each geologic region across Texas were the unit of analysis for exposure assessment.

Similar incidence rate ratios of any lymphoma were observed across areas with “medium-low”, “medium-high”, and the “highest” mean radon concentrations, compared to areas with the “lowest” mean concentrations: aIRR = 0.89, 95% CI: 0.79–1.00; aIRR = 0.90, 95% CI: 0.77–1.04; aIRR = 1.05, 95% CI: 0.83–1.32; *p*-trend = 0.35 (Table 4). Areas with “medium-low” radon concentrations had a significantly lower incidence of HL compared with areas of “lowest” radon concentrations (aIRR = 0.83, 95% CI: 0.71–0.98). However, this trend was not observed with increasing exposure (*p*-for-trend = 0.20). Areas with “highest” radon concentrations had a marginal but non-significant increase in non-BL NHL incidence compared to areas with the “lowest” radon concentrations (aIRR = 1.37, 95% CI: 0.95–1.97), however no trend was evident for this subtype (*p*-for-trend = 0.96). A similar pattern was seen for BL, where areas with “highest” radon concentrations had a marginal but non-significant increase in incidence compared to areas with the “lowest” radon concentrations (aIRR = 1.33, 95% CI: 0.70–2.53, *p*-for-trend = 0.54). Last, DLBCL incidence was increased in areas with the “highest” mean radon concentrations, although this trend was not identified with increasing exposure (aIRR = 1.73, 95% CI: 1.03–2.91, *p*-for-trend = 0.22). Notably, only three regions in Texas (Figure 1) were characterized as having “high” radon levels. Furthermore, the region with the highest levels (region 13) appeared to be driving the associations that were seen in that exposure category.

**Table 4:** Associations between indoor radon exposure and incidence rates of lymphoma malignancy subtypes among children and adolescents <20 years of age diagnosed in Texas, 1995–2011.

Lymphoma Subtype	Cases (*n*)	IR *	IRR * (95% CI)	aIRR * (95% CI)	*p*-for-Trend
*All Lymphomas*					
≤25th percentile (reference)	970	34.25	1.00	1.00	0.35
>25th to ≤50th percentile	711	30.92	0.88 (0.70–1.10)	0.89 (0.79–1.00)	
>50th to ≤75th percentile	373	33.38	0.98 (0.73–1.32)	0.90 (0.77–1.04)	
>75th percentile	93	33.88	0.93 (0.67–1.30)	1.05 (0.83–1.32)	
*Hodgkin Lymphoma*					
≤25th percentile (reference)	583	20.59	1.00	1.00	0.20
>25th to ≤50th percentile	391	17.00	0.86 (0.64–1.15)	0.83 (0.71–0.98)	
>50th to ≤75th percentile	227	20.32	0.99 (0.68–1.45)	0.94 (0.77–1.14)	
>75th percentile	47	17.12	0.81 (0.51–1.26)	0.87 (0.63–1.20)	
*NHL excluding BL*					
≤25th percentile (reference)	289	10.20	1.00	1.00	0.96
>25th to ≤50th percentile	231	10.04	0.96 (0.74–1.25)	1.00 (0.82–1.21)	
>50th to ≤75th percentile	103	9.22	0.92 (0.66–1.29)	0.82 (0.64–1.05)	
>75th percentile	35	12.75	1.26 (0.83–1.93)	1.37 (0.95–1.97)	
*Burkitt Lymphoma*					
≤25th percentile (reference)	98	3.46	1.00	1.00	0.54
>25th to ≤50th percentile	89	3.87	0.98 (0.65–1.48)	1.01 (0.73–1.38)	
>50th to ≤75th percentile	43	3.85	1.08 (0.64–1.80)	1.04 (0.71–1.52)	
>75th percentile	11	4.01	1.05 (0.52–2.14)	1.33 (0.70–2.53)	
*Diffuse Large B-Cell*					
≤25th percentile (reference)	123	4.34	1.00	1.00	0.22
>25th to ≤50th percentile	119	5.17	1.11 (0.80–1.55)	1.11 (0.85–1.44)	
>50th to ≤75th percentile	56	5.01	1.11 (0.74–1.68)	1.01 (0.73–1.39)	
>75th percentile	17	6.19	1.42 (0.81–2.49)	1.73 (1.03–2.91)	

CI, confidence interval; IR, incidence rate; IRR, incidence rate ratio; * Per 100,000 individuals; aIRR, adjusted for race/ethnicity, sex, category of age at diagnosis, and county-level SES/level of urbanization.

### 3.2. Discussion

In one of the largest studies of its kind, we found little evidence to suggest radon is positively or negatively associated with childhood lymphoma incidence. Further, while areas characterized as having the “highest” concentrations of radon did have a significantly higher incidence of DLBCL when compared to areas with the “lowest” concentrations of radon, a dose-response relationship was not observed for increasing exposure. While there have been few previous assessments of residential radon exposure and childhood lymphoma, our results are largely consistent with studies evaluating residential radon and childhood leukemia, where only weak associations have been suggested [26,28,42]. Like leukemia, lymphoma is considered to be a radiosensitive malignancy as it originates from mutated immune cells [32]. However, our findings do not suggest that residential levels of radon are strongly nor consistently associated with risk of this malignancy in children.

Radon is a radioactive decay product of uranium, and occurs naturally in soil, bedrock, and groundwater [43,44]. This inert, odorless gas migrates from the soil and penetrates buildings through cracks when a difference between soil gas and indoor air pressure exists [44,45]. An established public health concern, this carcinogenic, radioactive decay product is responsible for more than half of an individual’s average annual radiation dose, even though it remains undetected to those exposed [22,46,47,48]. Indoor radon exposure is the second leading cause of overall lung cancer, and the leading cause of lung cancer among never-smokers [45,49,50]. Furthermore, radon-associated lung cancer in never-smokers is also characterized by a younger age of onset [51]. While the risk of adverse health effects increases with higher dosage and longer durations of exposure, measuring the impact of low-level radiation levels remains difficult [44,46]. Despite this challenge, elucidating the role of domestic radon exposure in tumorigenesis continues to be of public health importance and interest. As radon decay products lead to DNA damage, somatic mutations and chromosomal aberrations, investigating an association with a malignancy such as lymphoma, which is influenced by genetic abnormalities, is well-founded [22,52,53].

The majority of studies evaluating the role of radon on lymphoma outcomes have focused on adults. One of the few studies to investigate radon and childhood lymphoma, a case-control study from Denmark between 1968–1994, determined a null association in relation to domestic radon exposure (lymphoma relative risk (RR): 0.90, 95% CI: 0.62–1.36) [30], which is similar to our findings. In one recent study among adults in the Eldorado uranium workers cohort, exposure to radon decay products was associated with a modest, but non-significant excess relative risk (ERR) in the incidence of HL among a small case group (HL ERR per 100 working level months: 20.7, 95% CI: 0.00–324.00, *p*-value: 0.08) [33]. A second study investigating this association among Czech uranium miners concluded that incidences of HL and NHLs were not increased due to radon exposure (HL RR: 2.12, 95% CI: 0.81–5.52; NHL RR: 0.80, 95% CI: 0.46–1.37) [54].

Our results did suggest that incidence of HL was marginally lower in areas of “medium-low” exposure. As relevant risk factors for HL in early life remain elusive, this may represent a previously unidentified association [15]. However, as a lack of biologic plausibility is present, there is also the possibility that this observation may be due to residual confounding, or related to unknown confounders, rather than an inverse association between radon exposure and HL incidence. Our results further suggested a modest increase in DLBCL incidence for areas with the “highest” mean radon concentrations compared to areas of the “lowest”. This may be a true association in support of a threshold effect for this lymphoma subtype. However, we cannot rule out that this increase in incidence may have been driven by the limited sample size of this strata (*n* = 17 DLBCL cases in this “highest” category) [55], especially as a trend with increasing exposure was not evident. Thus, these results in support of an association between residential radon and either childhood HL or DLBCL incidence remains non-conclusive.

There are certain limitations associated with the exposure assessment. First, the Texas Indoor Radon Survey utilized activated charcoal adsorption canisters rather than alpha track detectors. However, this was done largely in an attempt to capture exposure throughout the state in a cost-effective manner [34]. Second, there was no seasonal assessment of radon levels. Specifically, measurements were taken only in winter months when levels are highest. Therefore, we categorized exposure assuming that while levels may change, the relative ranking of regions would remain comparatively consistent. Third, radon exposure was measured in a centrally-located interior room as opposed to a bedroom where inhabitants may spend the most of their time while home, and thus may not have captured the area in which the main radon exposure may have occurred. Radon levels however are not likely to fluctuate greatly inside a single home, except for a potential decrease in radon concentrations in higher levels of a house (e.g., second-level of a house compared to the basement) [56]. Finally, the data generated from the Texas Indoor Radon Survey suggested that radon levels across the state of Texas are moderately low, with particular “hot-spots”, such as the Texas Panhandle, in concordance with the geologic substructure. This in-turn decreased the range of mean radon concentrations for analytic comparison resulting in a limited number of regions categorized as experiencing the “highest” levels of exposure.

Our study must be considered in the light of additional limitations. As our study was ecologic in nature, one potential limitation was the use of an area-level exposure assessment, which may have resulted in exposure misclassification [57]. However, population-scale individual-level measurements of radon exposure are nonexistent, and monitoring of radon in communities throughout the US is very limited. Therefore, the data generated through the Texas Indoor Radon Survey provide an important and cost-effective resource to evaluate the question of whether residential radon is associated with childhood lymphoma. Similar data sources and ecologic assessments have contributed a great deal of knowledge in terms of elucidating the association between residential radon and cancer [31,42,58,59,60,61,62,63]. Furthermore, county-level and area-level based estimates of residential exposures are commonly used when evaluating environmental contributions to disease [64,65,66,67]. In fact, data from the Texas Indoor Radon Survey have been used recently to explore the association between residential radon levels and structural birth defects [35].

Another potential limitation is exposure misclassification due to residential mobility during critical periods of development and windows of exposure. Specifically, as we only had access to residential information at diagnosis, we were not able to capture changes in exposure for those who may have moved between birth and diagnosis. While mobility during this time is likely, recent studies of the impact of residential mobility on environmental exposure assessment indicate individuals do not typically move great distances, most often staying within the same county or region [68,69,70]. This may lessen the impact of residential mobility on exposure misclassification in our assessment. However, we acknowledge that residential mobility may impact our exposure assessment and our findings.

Last, our exposure of mean radon concentration was estimated in 1991 while our case population was diagnosed between 1995 and 2011. We believe this may minimally influence our results as radon production levels should have remained constant over the period of 1995–2011 [35]. Radon decay products are in equilibrium with underground radium-226 whose half-life is 1600 years [45,47]. Thus, while radon levels over this time period were unlikely to have changed given the length of this half-life, methods of housing construction to influence indoor radon concentration may have changed during this time period [35,71]. A main and probable change in housing construction over this time period would include improved methods of insulation that may have promoted the containment of radon and its progeny [72,73]. Levels of radon exposure may thus have been greater than what was actually captured by the survey instrument resulting in an underestimation of effect size. However, despite potential changes in housing construction, building materials, and other unknown factors that may have influenced radon levels at the time of the survey, we are confident that the measurements from 1991 are reasonable proxies for approximate radon levels over the time of case diagnoses. This conclusion is further supported by the fact that over half of our case population (55.5%) was alive during the time that the exposure assessment was conducted.

A primary strength of our study was the inclusion of over 15 years of data for the state of Texas, with a relatively robust sample size of over 2000 childhood lymphoma cases representing one of the largest studies of this malignancy in children [74,75,76]. Given this reasonably large sample size, we were able to stratify by subtype to comprehensively evaluate our exposure in relation to the most prevalent lymphoma types, which are heterogeneous in nature [77]. Additional strengths of our study included the utility of a population-based sample, which limits the potential for selection bias as none of the study subjects self-selected to participate [65]. Further, we assessed our population and case estimates, in particular SES and level of urbanization, at the county-level prior to additional aggregation, as utilizing a smaller unit of analysis is one method of bias reduction when investigating aggregate data [57,78]. An added benefit of an area-based socioeconomic measure for confounder control is that it represented a mix of both individual-level and area-based socioeconomic effects to diminish potential bias [79]. Last, as we investigated childhood cancers with latency periods that are shorter compared to those of adult malignancies, limiting the possibility for unmeasured confounders or other methodological issues oftentimes related to the constructing exposure histories for adult cancers [80,81,82,83].

## 4. Conclusions

In conclusion, we found little evidence to suggest residential radon exposure is either positively or negatively associated with childhood lymphoma incidence; similar to previous studies evaluating this environmental exposure and other malignancies in children such as leukemia. To our knowledge, this is the first epidemiologic evaluation of residential radon exposure and childhood lymphoma in Texas, and one of few studies to investigate this exposure in regards to lymphoma among those less than 20 years of age. Future assessments should consider finer levels of exposure assessment, potentially through the use of alpha-track detectors, in order to fully evaluate the potential for residential radon exposure to impact childhood lymphoma incidence. Further, the use of alternative study designs, such as incorporating pooled data from multiple studies, could be utilized to increase the study power of each lymphoma phenotype and add to the clarification of any association. Last, incorporating biomarkers of exposure and response may also significantly improve the potential to further elucidate any association between residential radon and childhood cancers such as lymphoma.
